# Cysteine Methylation Controls Radical Generation in the Cfr Radical AdoMet rRNA Methyltransferase

**DOI:** 10.1371/journal.pone.0067979

**Published:** 2013-07-05

**Authors:** Martin R. Challand, Enrico Salvadori, Rebecca C. Driesener, Christopher W. M. Kay, Peter L. Roach, James Spencer

**Affiliations:** 1 School of Cellular and Molecular Medicine, University of Bristol Medical Sciences Building, Bristol, United Kingdom; 2 Institute of Structural and Molecular Biology, University College London, London, United Kingdom; 3 London Centre for Nanotechnology, University College London, London, United Kingdom; 4 Chemistry, University of Southampton, Highfield, Southampton, United Kingdom; 5 Institute for Life Sciences, University of Southampton, Highfield, Southampton, United Kingdom; The Scripps Research Institute, United States of America

## Abstract

The ‘radical *S*-adenosyl-L-methionine (AdoMet)’ enzyme Cfr methylates adenosine 2503 of the 23S rRNA in the peptidyltransferase centre (P-site) of the bacterial ribosome. This modification protects host bacteria, notably methicillin-resistant *Staphylococcus aureus* (MRSA), from numerous antibiotics, including agents (e.g. linezolid, retapamulin) that were developed to treat such organisms. Cfr contains a single [4Fe-4S] cluster that binds two separate molecules of AdoMet during the reaction cycle. These are used sequentially to first methylate a cysteine residue, Cys338; and subsequently generate an oxidative radical intermediate that facilitates methyl transfer to the unreactive C8 (and/or C2) carbon centres of adenosine 2503. How the Cfr active site, with its single [4Fe-4S] cluster, catalyses these two distinct activities that each utilise AdoMet as a substrate remains to be established. Here, we use absorbance and electron paramagnetic resonance (EPR) spectroscopy to investigate the interactions of AdoMet with the [4Fe-4S] clusters of wild-type Cfr and a Cys338 Ala mutant, which is unable to accept a methyl group. Cfr binds AdoMet with high (∼ 10 µM) affinity notwithstanding the absence of the RNA cosubstrate. In wild-type Cfr, where Cys338 is methylated, AdoMet binding leads to rapid oxidation of the [4Fe-4S] cluster and production of 5'-deoxyadenosine (DOA). In contrast, while Cys338 Ala Cfr binds AdoMet with equivalent affinity, oxidation of the [4Fe-4S] cluster is not observed. Our results indicate that the presence of a methyl group on Cfr Cys338 is a key determinant of the activity of the enzyme towards AdoMet, thus enabling a single active site to support two distinct modes of AdoMet cleavage.

## Introduction

The radical *S*-adenosyl-L-methionine (AdoMet) enzyme Cfr is one of a growing number of enzymes that have been discovered to catalyse methyl transfer reactions to unactivated C–H bonds [1]. Cfr methylates the unreactive C2 and C8 carbon atoms of adenosine 2503 (A2503; *Escherichia coli* numbering) of the 23S RNA component of the large subunit of the bacterial ribosome (rRNA) [2], [3]. Methylation at C8 protects Cfr-producing bacteria from the action of a variety of antibiotics, including the oxazolidinone linezolid, pleuromutilins and chloramphenicol, that interact with the peptidyltransferase site (P-site) of the bacterial ribosome [4]. The *cfr* gene is carried on mobile genetic elements that have facilitated the rapid dissemination of this antibiotic resistance mechanism throughout the bacterial community including, since 2007, strains of methicillin resistant *Staphylococcus aureus* (MRSA) [5], and other Gram-positive and Gram-negative bacteria including Enterococci [6], [7] and *E. coli* [8]. The advent of transferable linezolid resistance is particularly significant as it highlights the availability of a pool of pre-existing resistance determinants to a fully synthetic agent with no natural product origin [9]. In most bacteria, including *S. aureus*, A2503 is methylated at the C2 position by a related enzyme, RlmN [10], from which Cfr is thought to have evolved. Recent investigations [11]–[14] have established that these enzymes achieve this challenging reaction by distinctive catalytic mechanisms.

Members of the radical AdoMet enzyme superfamily have attracted much recent interest as awareness grows of their wide distribution and the extensive range of biochemical reactions that they are able to catalyse ([15] and references therein). These enzymes contain a [4Fe-4S] cluster in the active site, usually ligated via three of the iron atoms to a conserved CXXXCXXC sequence motif. Structural analyses of several radical AdoMet enzymes [16]–[23] have demonstrated a common AdoMet binding mode where AdoMet binds via its amino acid moiety to the fourth iron atom of the [4Fe-4S] cluster (termed the ‘unique iron’; ([24] and references therein)) in a bidentate manner. This brings the sulfonium ion of AdoMet into close proximity with the cluster (typically between 3–4 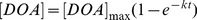). AdoMet binding in this manner raises the mid-point potential for a one electron reduction of the [4Fe-4S] cluster from an overall +2 to +1 oxidation state into the range where this can be facilitated by a physiological reducing agent (such as flavodoxin in *E. coli*) [25], [26]. Subsequent electron transfer from the reduced [4Fe-4S]^1+^ cluster to the juxtaposed sulfonium ion of AdoMet triggers homolysis of the adjacent 5′C-S bond to yield methionine and a highly reactive primary 5′-deoxyadenosyl radical (5′dA•). This potent oxidant can initiate a wide range of chemically challenging biological processes by abstracting a hydrogen atom from unreactive centres to generate a substrate radical. Substrates for radical generation range from small molecule precursors, or biosynthetic intermediates, for diverse primary and secondary metabolites [27], to proteins [28] and nucleic acids [29].

**Figure 1:**
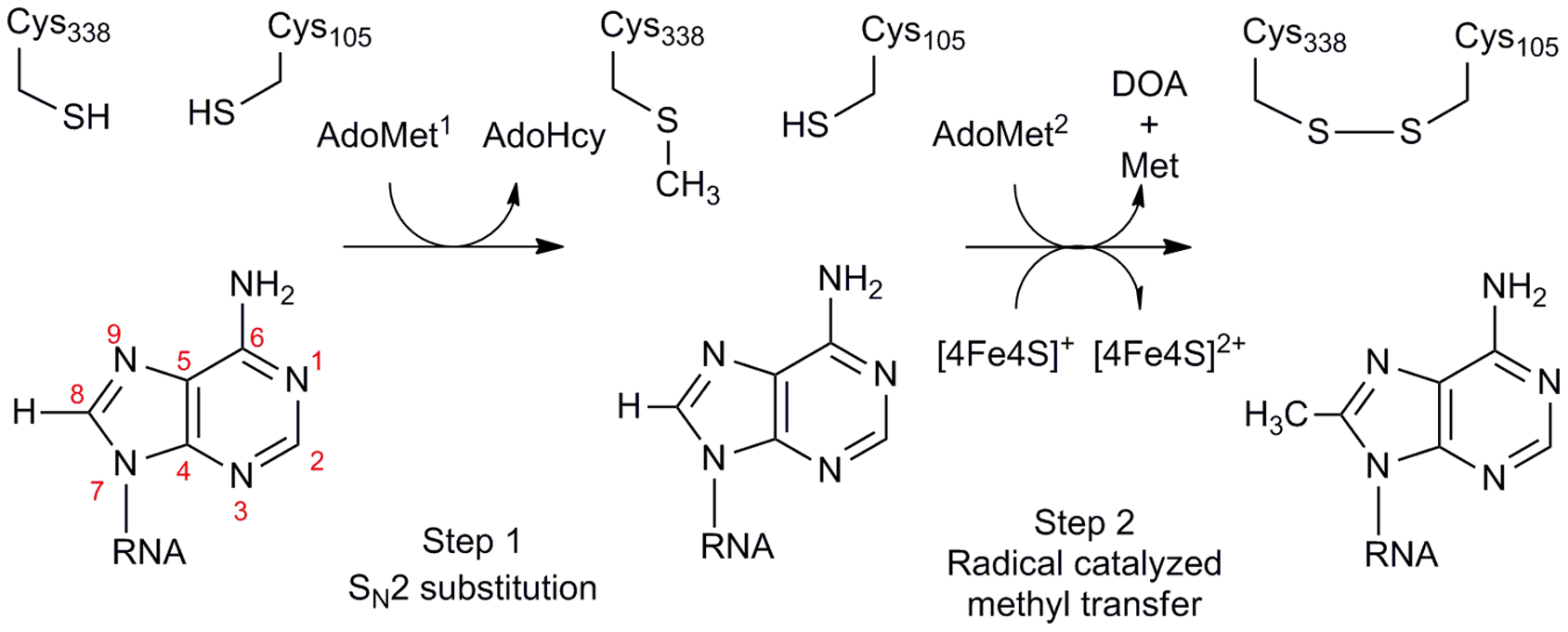
Utilisation of AdoMet by Cfr. Cfr consumes two AdoMet equivalents per reaction cycle to support both methyl transfer to Cfr Cys338 (AdoMet^1^; step 1) and subsequently generation of the 5′dA⋅ radical (AdoMet^2^; step2).

Recent reports [11]–[13], [18] have provided substantial mechanistic details for the methyl transfer reaction(s) catalysed by Cfr and RlmN. It is now established that each turnover requires two molecules of AdoMet, one serving as the source of the methyl group and the other of 5′dA• (Figure 1). Experimental evidence indicates that the methyl group is transferred from AdoMet to the 23S rRNA via a conserved cysteine residue (Cys355 in RlmN or Cys338 in Cfr), located within a flexible loop at the C-terminus of the polypeptide chain [18]. Recent spectroscopic investigations [14] demonstrate that both Cfr and RlmN contain a single [4Fe-4S] cluster. AdoMet bound to this cluster can both undergo reductive cleavage to 5′dA•, and react with the thiol moiety of (Cfr) Cys338 in an S_N_2 substitution reaction. The resulting methyl cysteine thioether (mCys) is then the substrate for reaction with 5′dA•, making Cfr and RlmN unusual radical AdoMet enzymes in that they catalyse hydrogen atom abstraction from a post-translationally modified residue of their own polypeptide chain.

Establishing how the single [4Fe-4S] cluster of these enzymes controls the turnover of AdoMet to permit two distinct reactivities (Figure 1, methyl transfer (step 1) and 5′dA• formation (step 2)) at specific points of the overall reaction cycle is then a central question in understanding the reactions of these mechanistically intriguing, and clinically important, enzymes. In pursuit of this goal we here use continuous-wave electron paramagnetic resonance (cw-EPR) and absorbance spectroscopy to study the [4Fe-4S] centres of wild-type Cfr and of a Cys338 Ala mutant (which is incapable of accepting a methyl group from AdoMet) in the presence and absence of AdoMet, and to determine dissociation constants for AdoMet binding. Our results show that AdoMet binding leads to rapid oxidation of the [4Fe-4S] cluster of wild-type Cfr and results in uncoupled cleavage of AdoMet to 5′-deoxyadenosine. However, in the Cys338 Ala Cfr mutant, which still possesses an intact [4Fe-4S] centre with spectroscopic properties similar to wild-type Cfr, AdoMet binding leads neither to oxidation of the [4Fe-4S] cluster nor to significant 5′-deoxyadenosine production. Our results indicate that methylation of Cfr Cys338 may be a prerequisite for generation of the 5′dA• and thus a means by which alternative reactivities towards AdoMet are controlled and uncoupled cleavage (in part) avoided.

## Materials and Methods

### Protein Expression

Recombinant Cfr was expressed in *Escherichia coli* by a modified version of our previously reported protocol [30]. The *cfr* open reading frame, fused to an N-terminal hexhistidine tag (His_6_-Cfr), was sub-cloned from plasmid pACYC:Cfr [30] into the Nde1 and Xho1 sites of the expression vector pCDFDuet-1 (Novagen) to create plasmid pMC115. pMC115 and plasmid pDB1282 (a pAra13 derivative encoding the *isc* operon of Fe-S cluster assembly proteins from *Azotobacter vinelandii*; [31]) were co-transformed into *E. coli* Rosetta2(DE3)pLysS (Merck Millipore) and plated on L-agar (Maitland) containing 34 µg/mL chloramphenicol, 50 µg/mL streptomycin and 100 µg/mL ampicillin. A single colony was used to inoculate a 50 mL starter culture in L-broth (Maitland) plus antibiotics as above, which was cultured overnight and used as a 1% inoculum for fresh L-broth (4×1 L). These bacterial cultures were incubated in an orbital shaker (150 rpm) at 37°C until absorbance at 600 nm reached approximately 0.3. At this point 10 mM arabinose was added to induce expression of the isc proteins and FeSO_4_.7H_2_O and L-cysteine added to final concentrations of 2 mM and 200 µM, respectively. Culture at 37°C was continued until absorbance at 600 nm reached approximately 0.8, at which point the flasks were cooled to 18°C, Cfr expression induced by the addition of IPTG (1 mM) and culture continued at 18°C for a further 12 h. Bacteria were then harvested by centrifugation (5,000 g, 30 min, 4°C), the supernatant decanted and the wet cell paste stored at −80°C until purification.

### Site Directed Mutagenesis

The Cfr Cys338 Ala mutant was prepared using the Quikchange Lightning site directed mutagenesis kit (Agilent) following the manufacturer’s protocol, using primers Cfr C338A Fwd (5′-caatttgggattgatattgacgctgctgctggtcaattatatggtaattatc-3′) and Cfr C338A Rev (5′-gataattaccatataattgaccagcagcagcgtcaatatcaatcccaaattg-3′). The presence of the mutation, and the absence of any secondary mutations, was confirmed by DNA sequencing (The Sequencing Service, University of Dundee, U.K). Expression and purification of Cfr Cys338 Ala followed identical procedures to those described for the wild-type enzyme.

### Anaerobic Protein Purification and Reconstitution

Anaerobic procedures were carried out in a glove box (Belle Technology, Weymouth, U.K.) in a nitrogen atmosphere within a laboratory maintained at a constant 20°C. Chromatography steps utilised an AKTAPrime Plus workstation (GE Life Sciences). An anaerobic cell lysate was prepared by suspending wet cell paste (0.3 g/L) in anaerobic buffer A (50 mM bis-Tris-propane pH 8.4, 20% (v/v) glycerol, 0.25 M NaCl, 20 mM imidazole). An EDTA free protease inhibitor tablet (Roche), Benzonase (New England Biolabs; ∼ 2,500 units)) and lysozyme (0.5 mg/mL) were added and the suspension stirred at r.t. for 30 min before cooling to ∼10°C in a chilled water bath. Cells were disrupted by sonication, transferred to a gas tight centrifuge bottle and the insoluble debris removed by centrifugation (15,000 g, 30 min, 4°C). The cleared supernatant was applied to a 20 mL chelating Sepharose column (GE Life Sciences), previously charged with NiSO_4_ and equilibrated with anaerobic buffer A. The column was washed with anaerobic buffer A until absorbance at 280 nm approached the baseline. Bound His_6_-Cfr was eluted on a 40 mL 20 mM –250 mM imidazole gradient followed by isocratic elution at 250 mM imidazole collecting 3 mL fractions. Cfr containing fractions (coloured brown) were pooled and immediately exchanged into anaerobic buffer C (50 mM bis-Tris-propane pH 8.4, 10% (v/v) glycerol, 0.5 M KCl, 5 mM DTT) by size exclusion chromatography (50 mL Sepharose G-50 column (GE Healthcare)). Progress of the purification procedure was monitored by SDS-PAGE.

Dithiothreitol (DTT; 5 mM) was added to purified Cfr (20–25 mL in buffer C, 1.5–2.5 mg/mL). After 15 min gentle stirring 4 molar equivalents of iron(III) chloride were added dropwise from a freshly prepared 10 mM stock solution. The resulting dark red solution was stirred for a further 15 min followed by dropwise addition of 4 molar equivalents of Na_2_S.9H_2_O, also from a freshly prepared 10 mM stock solution in anaerobic water. After 60–90 min the dark brown solution was centrifuged (15,000 g, 5 min, 4°C) to remove precipitated protein and excess FeS. Reconstituted Cfr was stored at −80°C and thawed anaerobically as required. Iron content (of samples desalted on 2.5 mL Sephadex G-25 columns (PD-10; GE Life Sciences)) was determined by the method of Fish [32] using FeSO_4_.7H2O as a standard. Protein concentrations were determined using the Bradford assay [33] using bovine serum albumin (BSA) as a standard.

### Mass Spectrometry

Samples were desalted on 50 µm POROS R2 resin (Applied Biosystems) prior to mass spectrometry. Mass spectrometry analysis was by flow injection on a MicroTOF-Q mass spectrometer (Bruker) using an electrospray source operated in positive-ion mode. Summed spectra were deconvoluted using the maximum-entropy module of the Bruker data analysis software suite.

### AdoMet Production

Expression of *E. coli* AdoMet synthetase was carried out as described previously [34] with slight modifications. Briefly, an overnight starter culture of *E. coli* DM22 (pk8) (generous gift of J. Broderick, Montana State University) in 2YT media (Oxoid) with oxytetracycline (10 µg/mL, LB/Oxytet) was used as a 1% inoculum into fresh LB/Oxytet media (5 L). After incubation at 37°C in an orbital shaker (180 rpm) for 10 h the cells were isolated by centrifugation (7,500 rpm, 30 min, 4°C) typically yielding 2 g/L of wet cell paste. A crude cell lysate was prepared by resuspending the cell paste (0.3 g/L) in buffer D (100 mM Tris pH 8.0, 1 mM EDTA, 50 mg/mL lysozyme), the mixture was then stirred at room temperature for 30 min and lysed by 10×1 min sonication cycles. The lysate was cleared by centrifugation (7,500 rpm, 20 min, 4°C) and stored as 1 mL aliquots at −80°C.

Enzymatic AdoMet synthesis was achieved using a previously described method [34] with minor modifications. Reactions (20 mL) were prepared by dissolving ATP (13 mM) (Melford Laboratories Ltd) in buffer E (100 mM Tris pH 8.0, 26 mM MgCl_2_, 50 mM KCl, 1 mM EDTA) before addition of acetonitrile (20% (v/v)) and L-methionine (10 mM). AdoMet synthetase crude cell lysate (1 mL, ∼ 30 mg crude protein, as estimated by Bradford assay) was then added and the reaction gently stirred at room temperature for 4–5 h. Reaction progress was monitored by reverse-phase thin-layer chromatography (3∶1 water:acetonitrile, 0.1% acetic acid; R_f_ values: AdoMet –0.25, 5′-methylthioadenosine (MTA) –0.40, adenine –0.55, ATP –0.93). The pH was adjusted to ∼ 7 by addition of 1% TFA (trifluoroacetic acid, ∼ 0.5 mL) and the mixture chilled on ice for 20 min before storage at −80°C.

The optimised purification protocol included acidification of the reaction mixture to ∼pH 5 by addition of 1% TFA (1–1.5 mL) and removal of precipitated polypeptides by centrifugation (7,500 rpm, 20 min, 4°C). The resulting supernatant was filtered through a 0.22 µm filter (Millipore) and 10 mL applied, in 20 mM TFA, to an 8 mL Source 15S cation exchange column (GE Healthcare Life Sciences) previously equilibrated with 100 mM TFA. The column was further washed with 20 mM TFA until absorbance at 280 nm returned to baseline. Elution was effected by a 10 mL gradient to 1 M TFA. AdoMet typically eluted as a single peak at between 0.7–0.9 M TFA. AdoMet-containing fractions were identified and confirmed by TLC (AdoMet R_f_ value 0.75 under these acidic conditions), pooled and lyophilized prior to storage at −80°C. Overall yield was 20–30% and purity confirmed by HPLC (showing <0.1% adenine and <1.5% MTA) and ^1^H NMR: (D_2_O, 400 MHz) δppm 2.25–2.44 (m, 2 H, H_β_), 2.92 (s, 0.1 H, (*R*,*S*) SMe), 2.96 (s, 3 H, (*S*,*S*) SMe), 3.40–3.50 (m, 1 H, H_γ_), 3.60–3.70 (m, 1 H, H_γ_), 3.85–3.99 (m, 3 H, H_α_, H_5′_), 4.48–4.54 (m, 1 H, H_4′_), 4.54–4.59 (m, 1 H, H_3′_), 4.78 (t, ^3^ *J = *4.50 Hz, 1 H, H_2′_), 6.12 (d, ^3^ *J = *4.00 Hz, 1 H, H_1′_), 8.40–8.41 (m, 2 H, H_2,8_). Integration of the distinct signals of the AdoMet-methyl groups at 2.96 ppm and 2.92 ppm, respectively, in the ^1^H NMR spectrum [35] estimated the ratio of (*S*,*S*) and (*R*,*S*) AdoMet as being typically ∼ 95% of the active, (*S*,*S*) diastereoisomer. Stock solutions of purified AdoMet for use in spectroscopic experiments were prepared in appropriate anaerobic buffers and concentrations estimated from UV absorbance at 260 nm (ε = 15,400 M^−1^ cm^−1^ [36], [37]).

### Cw-EPR Spectroscopy

Reconstituted Cfr was concentrated in a stirred pressure cell (Amicon) using a 10 kDa molecular weight cut-off membrane (Amicon), exchanged into buffer F (50 mM HEPES pH 8.1, 10% (v/v) glycerol, 0.5 M KCl), diluted to 5 mg/mL (125 µM) and aliquots frozen at −80°C. Samples for spectroscopy were thawed anaerobically, mixed with sodium dithionite (1 mM) and/or purified AdoMet (400 µM) and incubated at room temperature for 30 min. 150 µL was transferred to an EPR tube (Wilmad, U.K.) which was sealed with a rubber septum, removed from the anaerobic chamber and immediately frozen in liquid nitrogen. Samples were maintained at liquid nitrogen temperatures at all times prior to obtaining EPR spectra. EPR measurements used a Bruker EMXplus spectrometer operating at 9.4 GHz (X-band) equipped with a 4122SHQE resonator, with an Oxford Instruments ESR900 cryostat for measurements in the temperature interval 10–40 K. Spectra were acquired with a magnetic field sweep from 0 to 600 mT, a microwave power of 2 mW, a modulation amplitude of 0.5 mT and a modulation frequency of 100 kHz. Simulations of the cw-EPR spectra, to obtain the g-tensor principal components, were undertaken using the Easyspin toolbox [38] running in a Matlab (MathWorks, U.S.A.) environment.

### HPLC Assays of DOA and AdoHcy Formation

Reconstituted Cfr was concentrated in a stirred pressure cell (Amicon) using a 10 kDa molecular weight cut-off membrane (Amicon). To ensure complete methylation of the wild-type enzyme AdoMet (Sigma; 1 mM) was added and the sample incubated for 30 min at room temperature. At this point a 50 µL aliquot was removed for quantification of *S*-adenosyl homocysteine (AdoHcy) production using HPLC as described below. The mixture was exchanged into buffer G (50 mM Bis-Tris-Propane pH 8.4, 10% (v/v) glycerol, 0.5 M KCl) by desalting on a disposable Sephadex G-25 column (Nap-10; GE Life Sciences) to remove any small molecules (including FeS, DTT, AdoMet and AdoHcy). Activity assays (final volume 100 µL in buffer G) were prepared by mixing either wild-type Cfr or Cfr Cys338 Ala (12.5 µM) and enzymatically synthesised and purified AdoMet (500 µM). Assays were equilibrated at 37°C for 5 min before initiation by addition of sodium dithionite (1 mM). Reactions were stopped over a range of selected time points by addition of 20% (v/v) perchloric acid (5 µL). Precipitated protein was removed by centrifugation (benchtop microcentrifuge, 13,000 rpm, 5 min) and the supernatant immediately analysed by HPLC injecting 10 µL onto a Gemini C18 (4.6×250 mm 5 µM, 110 Å) reverse phase column (Phenomenex). The mobile phase was an initial 8 min isocratic elution of 2.5% acetonitrile in 20 mM ammonium acetate pH 6.2 followed by a linear gradient to 25% acetonitrile over 12 min. DOA and AdoHcy concentrations were estimated from peak areas calibrated against synthetic standards (1–100 µM).

The data for wild-type Cfr were fitted to a first order process:
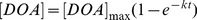
(1)where 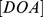 is the observed concentration of DOA; 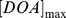 is the maximum observed DOA concentration, *t* is time and *k* is the observed first order rate constant. Under the conditions of the experiment (500 µM AdoMet), at *t* = 0, the Cfr will be saturated such that [Cfr] = [Cfr.AdoMet]. Therefore, the initial turnover number 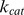 can be estimated from the following equation:
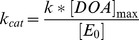
(2)where [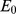] is the concentration of Cfr.

### Absorbance Spectroscopy

Absorbance spectroscopy was carried out in 1 cm cuvettes using an Ocean Optics (Duiven, The Netherlands) USB2000 spectrophotometer and Mini-D2-GS light source connected by optical fibres to a cuvette holder contained within an anaerobic chamber [39]. Experiments were carried out in buffer G. Small aliquots (≤10 µL) of purified AdoMet.TFA were titrated into Cfr samples (1 mL; 60 µM). Samples were gently mixed and equilibrated for 2 min prior to recording absorbance spectra. Data were corrected for dilution and fitted to the tight binding quadratic equation [40]:
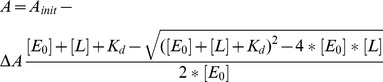
(3)Where 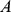, 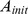 and 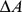 denote absorbance, maximal absorbance and maximal change in absorbance, respectively; 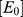 is the concentration of Cfr, 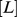 is the concentration of ligand and 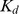 the dissociation constant.

## Results and Discussion

The procedures described yield purified recombinant Cfr in typical yields of 5 mg/L after reconstitution, with an iron content of 4.3+/−0.2 molar equivalents for the wild-type enzyme. We ascribe our previous suggestion that Cfr may contain more than one [4Fe-4S] cluster [30] to increased adventitious iron binding at the elevated pH (9.5) at which our previous protein purification was carried out. Purification and reconstitution under the less alkaline conditions (pH 8.4) reported here yields material with an iron content consistent with other recent reports [14].

Recombinant Cfr was further characterised to determine the methylation state of the reconstituted wild-type enzyme. Cfr expressed using the protocol described yielded an electrospray mass spectrum centred on a mass of 41,084.7 Da, compared to calculated masses of 41,073.96 Da for the unmethylated, and 41 087.99 Da for the fully methylated, protein. Observation of a mass between those of unmethylated and methylated Cfr, but closer to the value expected for the methylated form, indicates that the protein is isolated as a mixture of the two species in which the methylated form predominates.

The transfer of methyl groups from AdoMet to the acceptor residue (Cys338) of Cfr also results in the formation of the product AdoHcy. By using HPLC analysis to quantify the AdoHcy formed, the extent of Cys338 methylation in Cfr can be estimated. When reconstituted Cfr was incubated with excess AdoMet, and the formation of AdoHcy quantified by HPLC, 0.12+/−0.03 mole equivalents of AdoHcy were observed to accumulate. Consistent with recent publications [14], these data indicate that our expression, purification and reconstitution procedure yields wild-type Cfr with Cys338 predominantly (≥85%) methylated. On the basis of these results we consider that investigations of AdoMet association with wild-type Cfr are likely to report binding to an active site configured for reductive AdoMet cleavage and formation of the 5′dA• intermediate, and ultimately DOA product (Figure 1, AdoMet^2^_,_ step 2), rather than to an active site configured for methylation of Cys338 (Figure 1, AdoMet^1^_,_ step 1).

**Figure 2:**
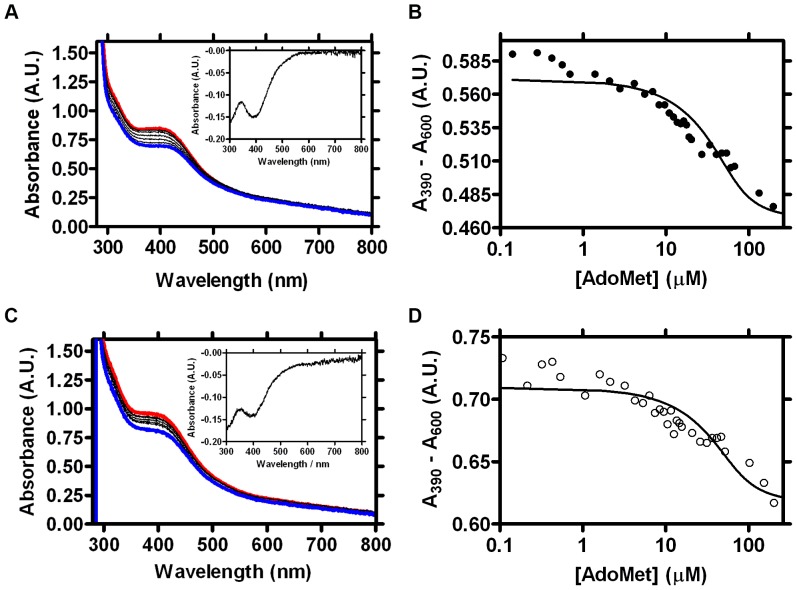
AdoMet Binding to the Cfr [4Fe-4S] Cluster. Interaction of AdoMet with the Cfr [4Fe-4S] cluster as monitored by absorbance spectroscopy. A. Absorbance spectra of reconstituted wild-type Cfr (60 µM; red trace) and on serial addition of AdoMet (black traces) to a final concentration of 260 µM (blue trace). Inset: difference spectrum obtained by subtracting last (blue; AdoMet) from first (red; no AdoMet) spectrum. B. Absorbance of reconstituted wild-type Cfr at 390 nm (corrected for baseline effects at 600 nm) plotted as a function of AdoMet concentration. Solid line is fit to equation 3 (R^2^ = 0.87) giving a dissociation constant of 8.4 µM. C. Absorbance spectra of reconstituted Cys338 Ala Cfr (60 µM; red trace) and on serial AdoMet addition (black traces) to 260 µM (blue trace). Inset: difference spectrum obtained by subtracting last (blue; AdoMet) from first (red; no AdoMet) spectrum. D. Absorbance of reconstituted Cys338 Ala Cfr at 390 nm (corrected for baseline effects at 600 nm) plotted as a function of AdoMet concentration. Solid line is fit to equation 3 (R^2^ = 0.83) giving dissociation constant of 11.7 µM.

We next used spectroscopic methods to investigate the [4Fe-4S] cluster of wild-type Cfr and its interactions with AdoMet. AdoMet purified from *in vitro* enzyme-catalysed syntheses (as described) was used in order to minimise the presence of contaminants arising from AdoMet racemisation and/or degradation [41]. Chemically reconstituted wild-type Cfr [30] was characterised by UV-Vis and cw-EPR spectroscopies. The visible absorbance spectrum of reconstituted Cfr has a broad peak around 400 nm, typical of intact [4Fe-4S] clusters ([42], [43]; Figure 1A) as found in radical AdoMet enzymes [44]–[47] and other iron-sulfur proteins [48]–[50]. Titration of AdoMet into this material causes a reduction in absorbance, with the resulting difference spectrum (Figure 2A, inset)) containing a clear feature around 390 nm. Addition of AdoMet has a similar effect upon the absorbance spectrum of the [4Fe-4S] cluster of biotin synthase [51] while crystal structures of AdoMet complexes of several radical AdoMet enzymes [24], including biotin synthase [17] and RlmN [18], show direct binding of AdoMet to the [4Fe-4S] cluster via bidentate coordination to the unique iron atom (a conformation favourable for reductive cleavage of the 5′C-S bond). We conclude that the observed absorbance changes are thus likely to result from a direct interaction between AdoMet and the [4Fe-4S] cluster of wild-type Cfr.

Observation of a measureable absorbance change upon AdoMet binding led us to investigate the concentration dependence of this spectroscopic signal. Monitoring absorbance at 390 nm during titration of wild-type Cfr with purified AdoMet (Figure 2B) produces a binding isotherm which could be fitted (equation 3) to yield a dissociation constant (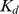) of 8.4 µM. (Note that, although use of a quadratic equation (equation 3) is mandated under these (tight binding) conditions, the experimental data did not well define the stoichiometry of the interaction and the enzyme concentration (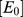; equation 3) was accordingly fixed at 60 µM as determined by Bradford assays). Interestingly, the measured value for 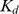 is consistent with that determined for AdoMet binding to biotin synthase in the presence (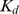 = 2.0 µM), but not the absence (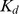 = 1 mM) of the co-substrate dethiobiotin [51]). This discrepancy may reflect the differing catalytic mechanisms of the two enzymes. In biotin synthase co-operative substrate binding (i.e. prior dethiobiotin binding as a prerequisite for high-affinity association of AdoMet) has been proposed to aid in preventing inappropriate generation of the 5′dA• and uncoupled turnover of AdoMet. In contrast, current mechanisms for Cfr and RlmN employ the 5′dA• radical to activate the Cys338-bound methyl group and form a protein methyl radical [13]. As the wild-type enzyme utilised for these experiments is predominantly methylated (as shown by mass spectrometry and SAH quantification experiments), the cosubstrate for the radical generation step (methylated Cys338) is already present and radical formation may proceed even (as here) in the absence of RNA.

**Figure 3:**
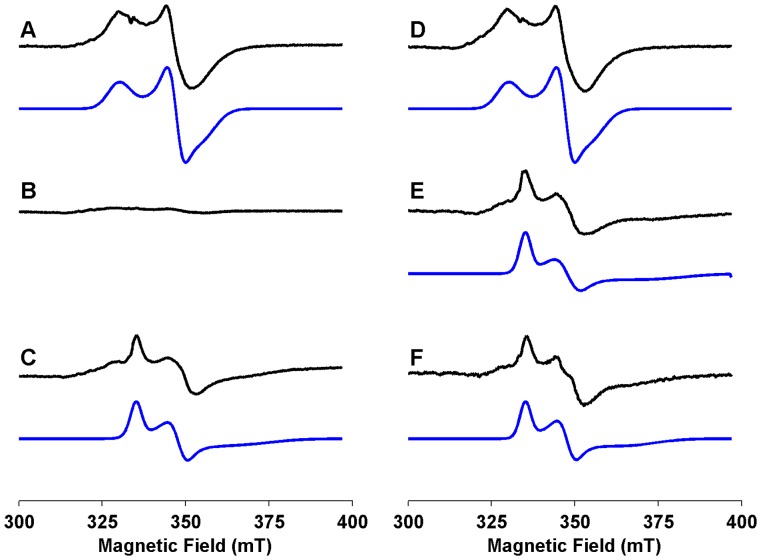
EPR Spectra of the Cfr [4Fe-4S] cluster. Cw-EPR spectra of wild-type Cfr (left hand column) and Cfr Cys338 Ala (right hand column). Experimental spectra are shown as black lines with corresponding simulations beneath (blue lines). A. Wild-type Cfr (105 µM) B. Wild-type Cfr (105 µM) plus AdoMet (300 µM). C. Wild-type Cfr plus AdoHcy (300 µM). D. Cfr Cys338 Ala (105 µM). E. Cfr Cys338 Ala plus AdoMet (300 µM). F. Cfr Cys338 Ala plus AdoHcy (300 µM). Note that all samples were reduced with sodium dithionite (1 mM).

To provide a more detailed description of the interaction of AdoMet with Cfr, cw-EPR spectroscopy was used to investigate the electronic structure of the Cfr [4Fe-4S] cluster and the effects upon it of interaction with AdoMet. Wild-type Cfr (105 µM) that had been reduced with an excess (10 molar equivalents) of sodium dithionite for 15 minutes yielded a nearly axial EPR spectrum (Figure 3A) with g values (Table 1) of 2.03(5), 1.93(2) and 1.89(0) (g_av_ = 1.95) indicative of a [4Fe-4S]^1+^ cluster. The temperature dependence of the signal [52] is consistent with the presence of a [4Fe-4S], rather than a [2Fe-2S], cluster: the spectrum reduces in intensity at temperatures higher than 30 K and is almost undetectable above 50 K (data not shown). Similar spectra have been reported for the [4Fe-4S] clusters of a number of other radical AdoMet enzymes [44], [45], [53], [54]. However, in the presence of an excess (3 molar equivalents) of AdoMet, we observed almost complete disappearance of the reduced [4Fe-4S]^1+^ signal (Figure 3B), implying that the cluster was most likely oxidised to [4Fe-4S]^2+^ under these conditions. This behaviour contrasts with that observed for many other radical AdoMet enzymes [34], [39], [44], [53], [55], [56], where, in the absence of cosubstrate, the EPR spectrum from the [4Fe-4S] cluster is maintained or even enhanced in the presence of AdoMet alone.

**Table 1:** g-Values for EPR Spectra of Cfr and Ligand Complexes.

	g1	g2	g3	g_av_
Cfr wild-type	2.03 (5)	1.93 (2)	1.89 (0)	1.95
Cfr wild-type+AdoMet	–	–	–	–
Cfr wild-type+AdoHcy	2.00 (0)	1.92 (6)	1.82 (3)	1.92
Cfr Cys338 Ala	2.03 (5)	1.93 (2)	1.89 (0)	1.95
Cfr Cys338 Ala+AdoMet	2.00 (0)	1.92 (5)	1.79 (0)	1.90
Cfr Cys338 Ala+AdoHcy	2.00 (0)	1.92 (7)	1.82 (3)	1.92

**Figure 4:**
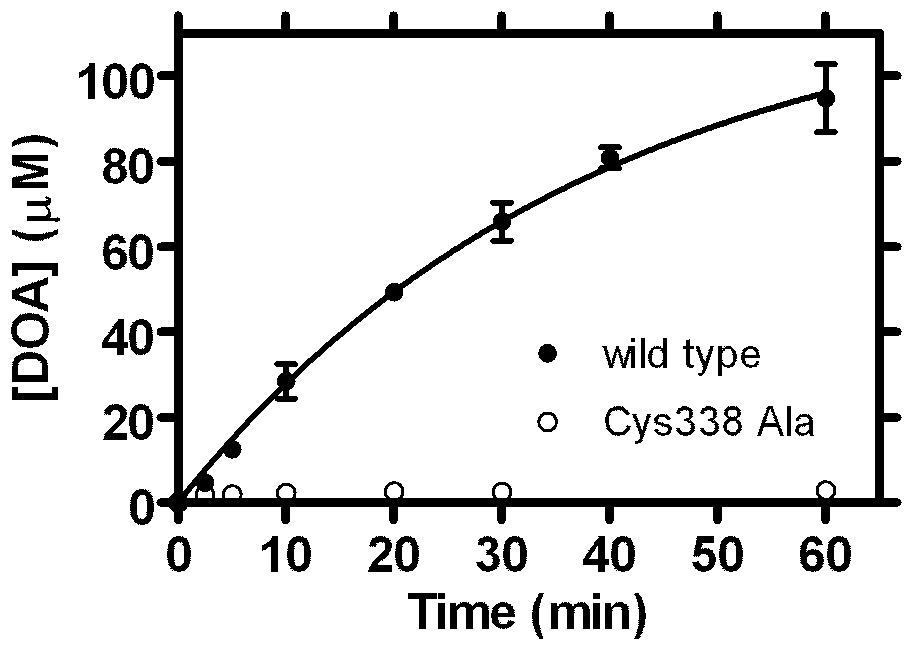
DOA production by Wild-type and Cys338 Ala Cfr. Production of 5′-deoxyadenosine (DOA) from uncoupled cleavage of AdoMet by wild-type (filled circles) or Cys338 Ala (open circles) Cfr assayed by reverse-phase HPLC. Solid line shows fit of wild-type data to equation 2 (*R^2^* = 0.99) yielding a *k*_cat_ value of 42×10^−4^ s^−1^. Data points are the means of two independent measurements of separately prepared samples, shown with their standard error.

These data indicate that wild-type Cfr is able to bind AdoMet with high affinity in the absence of the RNA cosubstrate, and that this probably leads to oxidation of the Cfr [4Fe-4S] cluster. The simplest explanation for this observed oxidation is that bound AdoMet undergoes reductive cleavage of the 5′C-S bond (Figure 1 step 2) consistent with previous observations [30] of uncoupled turnover of AdoMet to 5′-deoxyadenosine (DOA) by Cfr in the presence of sodium dithionite. Uncoupled turnover of AdoMet to DOA has been described for a number of other radical AdoMet enzyme systems [44], [57]–[59]. To further investigate this hypothesis, we used RP-HPLC analysis at selected time points to quantify the uncoupled formation of DOA. After 1 h incubation at 37°C, almost 10 molar equivalents of DOA had formed with respect to Cfr concentration (Figure 4). This time course could be fitted to a first order process (Equation 1) and an initial turnover number (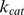) derived (Equation 2) of 42×10^−4^ s^−1^. The estimated value of 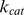 is comparable to those determined for a number of other class 3 radical AdoMet enzymes [15]. (Class 3 radical AdoMet enzymes utilise the 5′-deoxyadenosine radical stoichiometrically with respect to the number of hydrogen atom abstraction steps required during each enzyme turnover, and therefore generate DOA as a byproduct [60]).

It has recently been proposed that the single [4Fe-4S] cluster of Cfr (and RlmN) is able to bind AdoMet for both methyl transfer to Cys338 (Figure 1, step 1) and subsequent formation of 5′dA• ([14]; Figure 1, step 2). Understanding how this is achieved requires description of the interaction(s) of AdoMet with Cfr in the presence and absence of a methyl group on Cys338. However, investigation of AdoMet binding to the [4Fe-4S] cluster of unmethylated Cfr is complicated by the difficulty of isolating the enzyme in a non-methylated state [14] and the ability of the enzyme to rapidly methylate on exposure to AdoMet. Accordingly, we used the methylation-incompetent Cys338 Ala mutant [14] as a system in which to study the interaction of AdoMet with the [4Fe-4S] cluster of the unmethylated enzyme (Figure 1 step 1). Cys338 Ala Cfr was expressed, purified and reconstituted using identical methodologies to the wild-type enzyme, and incorporates similar quantities of iron. To investigate the catalytic properties of Cys338 Ala Cfr, we used HPLC analysis to quantify production of AdoHcy and DOA when the AdoMet substrate was present in excess under reducing conditions. In contrast to the wild-type enzyme, Cfr Cys338 Ala was unable to catalyze production of AdoHcy or DOA, even after extended incubation in the presence of sodium dithionite (After 1 hour incubation, the amount of DOA produced by Cfr Cys338 Ala is 33-fold less than that obtained with the wild-type enzyme (Figure 4)). These data confirm that Cys338 Ala Cfr is unable to accept a methyl group from AdoMet, and that the formation of 5′dA• is significantly impaired.

The Cfr Cys338 Ala mutant was then subjected to spectroscopic investigation in order to assess the impact of the mutation upon the [4Fe-4S] centre and its interaction with AdoMet. The visual absorbance spectrum of the reconstituted mutant enzyme (Figure 2C) closely resembles that of wild-type Cfr, consistent with presence of an intact [4Fe-4S] cluster. Furthermore, the response of this mutant to titration with AdoMet (Figure 2C) is also very similar to that of the wild-type enzyme (above; Figure 2A) with a saturable reduction in visible absorbance generating a difference spectrum with a prominent feature around 390 nm. As for wild-type Cfr, these data were fitted to yield a dissociation constant for AdoMet binding of 11.7 µM (Figure 2D). On this basis we conclude that the Cys338 Ala mutation, and by implication the presence of a methyl group upon Cys338, has little effect on the affinity of Cfr for AdoMet.

While absorbance spectroscopy demonstrates that Cys338 Ala Cfr contains a [4Fe-4S] cluster that retains the ability to bind AdoMet, this does not reveal whether the mutation introduces changes to the structure or electronic properties of the cluster. To address this question cw-EPR spectroscopy was used to investigate the electronic structure of the [4Fe-4S] cluster of Cys338 Ala Cfr in the presence and absence of AdoMet. The EPR spectrum of chemically reconstituted and reduced Cys338 Ala Cfr (Figure 3D) closely resembles that of wild-type Cfr (g-values 2.03(5), 1.93(2) and 1.89(0); Table 1), indicating that the absence of the methyl group on Cys338) does not substantially alter the co-ordination environment of the [4Fe-4S] cluster. However, in contrast to the wild-type enzyme, reduction of Cfr Cys338 Ala in the presence of AdoMet (3 molar equivalents) gave an observable, although weaker, EPR spectrum (Figure 3E). Binding of AdoMet to the [4Fe-4S] cluster induces significant changes in the EPR spectrum: the signal symmetry becomes more rhombic with g-values 2.00(0), 1.92(5) and 1.79(0) (g_av_ = 1.90). The principal change is in the g3 value, which decreases from 1.89 to 1.79 on AdoMet binding, indicating a possible loss of symmetry in the cluster environment. These data indicate that direct interaction with AdoMet can perturb the electronic structure of the Cys338 Ala Cfr [4Fe-4S] cluster, but does not lead to its oxidation. The spectrum obtained in the presence of AdoMet to some extent resembled those observed for some other radical AdoMet enzymes (such as viperin, anaerobic ribonucleotide reductase and anaerobic sulfatase-maturating enzyme [44], [53], [56]) when AdoMet is co-ordinated to the [4Fe-4S] cluster. In these systems, presence of the second substrate is a pre-requisite for oxidation of the cluster in the presence of AdoMet and concomitant AdoMet cleavage.

As a further investigation of the effect of Cys338 methylation on the Cfr [4Fe-4S] cluster and its interactions with ligands, the EPR spectra of reduced wild-type and Cys338 Ala Cfr were measured in the presence of AdoHcy (Figure 3C;F). These experiments yielded near-identical EPR spectra, which most closely resembled those of Cys338 Ala Cfr in the presence of AdoMet (Figure 3E, for g-values see Table 1). This experiment confirms that, as well as exerting little effect upon the electronic properties of the Cfr [4Fe-4S] cluster, the Cys338 Ala mutation does not fundamentally disrupt the manner in which Cfr binds ligands. Thus the differences in observable properties of wild-type (methylated) and Cys338 Ala (non-methylated) Cfr described above reflect specific effects upon interactions with AdoMet rather than a general alteration of the ligand-binding properties of the enzyme.

**Figure 5:**
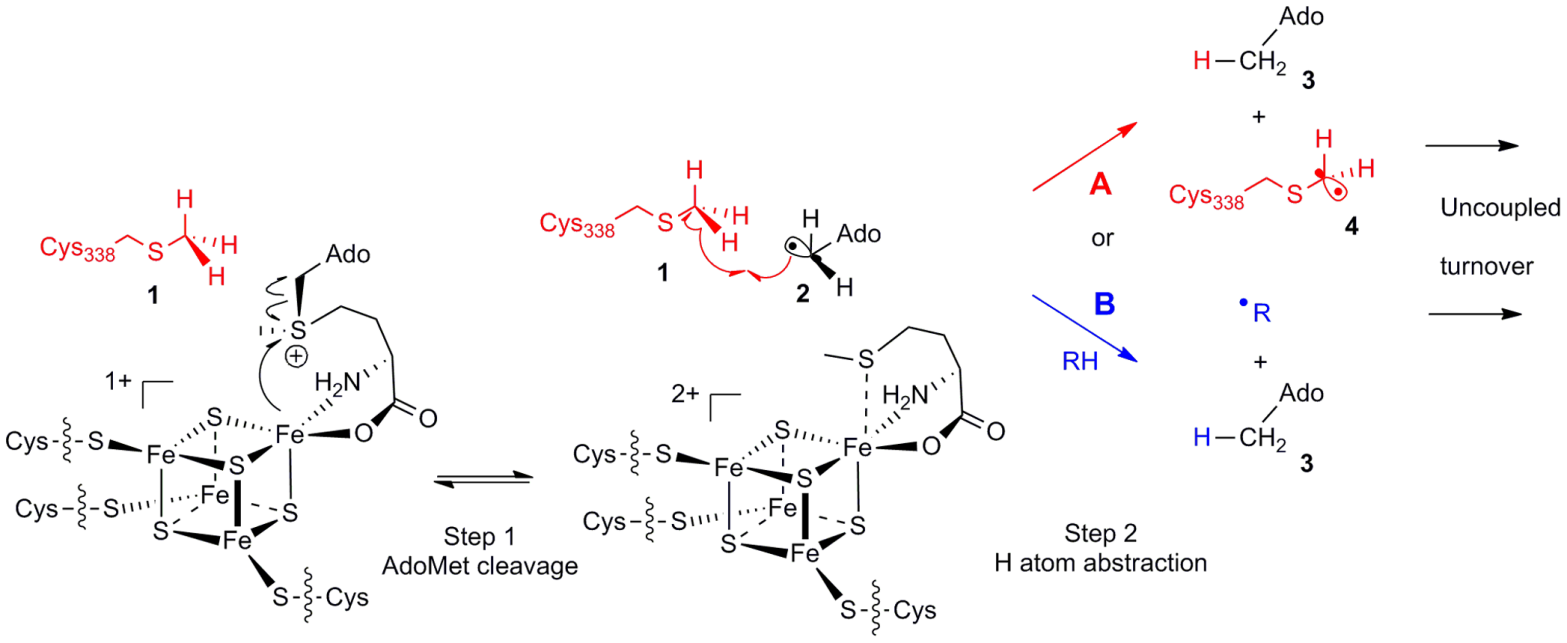
Mechanisms for Radical Generation and Uncoupled AdoMet Turnover by Cfr. Oxidative cleavage of AdoMet generates 5′dA⋅, **2**, which abstracts a hydrogen atom to yield the detected 5′-deoxyadenosine product, **3**. Uncoupled formation of 5′-deoxyadenosine could occur by quenching of 5′dA⋅ through abstraction of a hydrogen atom from either i) mCys338, **1**, yielding the thermodynamically favourable radical intermediate **4** (pathway A, shown in red) or ii) a non-specific functional group (pathway B, shown in blue). Further details are given in the text.

Taken together, these data support the hypothesis that AdoMet binds to the [4Fe-4S] cluster of Cfr (and by implication the related RlmN enzyme) for two distinct activities: methyl transfer to Cys338 (or Cys355 in RlmN; Figure 1 step 1) and formation of 5′dA• (Figure 1 step 2; Figure 5 step 1). The EPR spectra presented here support a model in which the absence of a methyl group on Cys338 neither significantly perturbs the Cfr [4Fe-4S] cluster nor affects its affinity for AdoMet. However, only when mCys338 is present does AdoMet binding lead to re-oxidation of the [4Fe-4S] cluster, reductive cleavage of AdoMet and ultimately to DOA formation. Under the experimental conditions described (1 mM sodium dithionite), the absence of the mCys residue severely impairs reductive cleavage of AdoMet and the [4Fe-4S] cluster remains in the reduced, +1, oxidation state. One possible explanation for these observations is that the presence of the mCys residue may provide additional steric constraints on bound AdoMet, compared to its positioning in the unmethylated active site, that orient it more effectively for reductive cleavage to 5′dA•. Imposition of a conformation that forces the sulfonium of AdoMet into closer proximity to the [4Fe-4S] cluster may facilitate the necessary electron transfer due to increased orbital overlap with the 5′C-S σ* orbital, presumably resulting in a lower activation energy for AdoMet cleavage. As the rate of electron transfer may be very sensitive to the interatomic distance and the overlap of the electron donor and acceptor orbitals, even small adjustments in atomic positions, such as those necessary to accommodate the thioether methyl group of methylated Cys338, may exert significant effects. In the crystal structure of the RlmN:AdoMet complex (where Cys355 is methylated) the AdoMet sulfonium approaches to 3.2 Å from the unique Fe atom of the [4Fe-4S] cluster [18], in an orientation resembling those observed in ternary (enzyme:AdoMet:substrate) complexes of other radical AdoMet enzyme systems [16], [17], [21], [23], [24].

Hydrogen abstraction by 5′dA• from mCys338 has been characterised as thermodynamically favourable [61] by comparison of bond dissociation energies for a methyl thioether (HS-CH_2_-H = 397 kJ/mol; as required to generate the radical, **4**, on Cfr mCys338, **1**, (Figure 5)) compared to a primary aliphatic group (R-CH_2_-H = 440 kJ/mol; as involved in interconversion of 5′dA•, **2**, and DOA, **3**). In the light of these thermodynamic data, it is then tempting to speculate that reductive cleavage of AdoMet by Cfr leads to hydrogen atom abstraction from mCys338, **1**, (Figure 5 step 2). This hypothesis requires the observed uncoupled turnover to proceed via the methyl thioether radical, **4** (Figure 5 step 2, A; an intermediate in the catalytic mechanism proposed by Grove *et al* [13]) rather than by direct quenching of 5′dA• by a reaction with an alternative (non-specific) agent (Figure 5 step 2, B). Thus, mCys338 may be considered to fulfill a dual role, both providing a steric constraint that orients bound AdoMet for reductive cleavage and an appropriate target for the 5′dA• so generated. Using the methods employed here we could not however confirm the presence of the methyl thioether radical as, under these conditions, the EPR signal of the reduced [4Fe-4S] cluster of wild-type Cfr was completely quenched within 90 seconds of AdoMet addition (data not shown). The RlmN crystal structure [18] shows that this putative radical intermediate would lie in close proximity to the solvent accessible active site cleft and may be subject to rapid quenching by stepwise reduction and protonation. This may explain why it is not readily spectroscopically observable. Use of stoichiometric quantities of reductant and/or rapid quenching techniques may be required to confirm the presence of this radical intermediate.

The results reported here also highlight significant differences between Cfr and RlmN and many other radical AdoMet enzymes. In the majority of such enzymes studied to date, 5′dA• initiates catalysis by abstracting a hydrogen atom directly from the second substrate. In some systems, presence of the second substrate is necessary for high affinity AdoMet binding [51] and reductive cleavage [62]–[64], providing a mechanism by which these enzymes avoid uncoupled formation of the reactive 5′dA•. In contrast, here high-affinity AdoMet binding to Cfr is observed notwithstanding the absence of the RNA cosubstrate, and the rate of DOA formation (42×10^−4^ s^−1^) is comparable to reported *k*_cat_ values for product formation by other radical AdoMet systems ([15] and references therein). Cfr and RlmN are unusual examples of radical AdoMet enzymes insofar as 5′dA• abstracts a hydrogen atom from a functional group covalently linked to its own polypeptide, rather than directly from a second substrate, in this case rRNA. In other systems (e.g. pyruvate formate lyase [23]) productive AdoMet binding requires the second substrate to be present, as shown by the substantial reorientation (a reduction in the AdoMet sulfonium:unique Fe distance from 6.1 Å to 3.2 Å) and ordering of AdoMet in ternary, as opposed to binary, complexes. Our spectroscopic investigations of Cfr instead indicate that, while mCys338 is required for reductive AdoMet cleavage, absence of the methyl group does not affect either the electronic structure of the [4Fe-4S] centre or its interactions with ligands (AdoHcy) as shown by the close resemblance of cw-EPR spectra of the wild-type and Cys338 Ala enzymes, both unliganded (Figure 3A and 3D) and in the presence of AdoHcy (Figures 3C and 3F). These data are consistent with the earlier conclusion that a single AdoMet binding site can support two distinct activities [14], and demonstrate that methylation has little effect upon active site conformation. The implication is that the (presumably subtle) conformational changes induced by the methylation of Cys338 in Cfr yield a similar change in reactivity towards AdoMet to that induced by secondary substrate binding to other radical AdoMet enzymes [23].

The observed behaviour of Cfr also differs from that reported for the related methyltransferase RlmN. In contrast to the results reported here for Cfr, Grove *et al*. show that mutation to Ala of the Cys338 equivalent, Cys355, in RlmN reduces, but does not abolish, the ability of the enzyme to catalyze uncoupled AdoMet cleavage in the presence of non-physiological reductant (sodium dithionite) [13]. Although no Cfr crystal structure is available, comparison with that recently determined for RlmN [18] indicates that Cys338 is located on a mobile loop close to the C-terminus of the protein. While the C-terminus of RlmN is formed by an extended α-helix, this helix is absent from Cfr, potentially increasing the mobility of the polypeptide chain in the vicinity of Cys338. Increased flexibility in this region may be required to enable alternative modes of RNA binding that permit Cfr to catalyse methyl transfer onto two positions, C2 and C8 of A2503, which lie on different sides of the adenosine ring system (Figure 1). The failure of the Cfr Cys338 Ala mutant to support AdoMet cleavage may then reflect local, but functionally significant, conformational differences between the two enzymes. More detailed structural and kinetic studies of both enzymes will be necessary to resolve this issue.

## Concluding Remarks

Radical AdoMet enzymes are now recognised to employ a wide variety of mechanisms to modify a diverse range of (usually unreactive) substrates. This diversity extends to methyl transfer reactions, that are now believed to be achieved by a variety of mechanisms [65]. Our work here shows that use by Cfr and RlmN of a methylated cysteine residue not only permits methyl transfer to intrinsically unreactive positions on the RNA substrate but also enables them to control the timing of radical generation in the catalytic cycle, making possible the use of a single active site to achieve two distinct reactions.
